# Evaluation of different machine learning methods for ligand-based virtual screening

**DOI:** 10.1186/1758-2946-3-S1-P41

**Published:** 2011-04-19

**Authors:** R Kurczab, S Smusz, AJ Bojarski

**Affiliations:** 1 Department of Medicinal Chemistry, Institute of Pharmacology Polish Academy of Sciences, Krakow, 31-343, Poland

## 

*In silico* High Throughput Screening of large compound databases has become increasingly popular technology of finding valuable drug candidates, by applying a wide range of computational methods, such as machine learning [1]. In recent years, many comparative studies of different machine learning methods performance in ligand-based virtual screening have been reported [2,3].

In order to extend these studies, we have evaluated over 60 different machine learning methods, such as: support vector machines (with and without parameter optimization), naïve Bayesian, decision trees, random forest, meta-classifiers (boosting, bagging, grading) and many others. All calculations were performed using a collection of machine learning algorithms for data mining implemented in WEKA package [4]. Additionally, for each of the method, we have examined the influence of different type of fingerprints, the size of training sets and attribute selection methods on the rate of active recall and precision of selection. Our internal database of known 5-HT7 antagonists has been used to build training and testing sets.

It was found that there is no machine learning approach that consistently provides the best results but some of them are very stable and can be applied universally.
